# Dissemination and Continuous Improvement of a CTSA-based Software Platform, SPARCRequest©, Using an Open Source Governance Model – ADDENDUM

**DOI:** 10.1017/cts.2019.438

**Published:** 2020-04

**Authors:** Wenjun He, Royce Sampson, Jihad Obeid, Kyle Hutson, Boyd M. Knosp, Bernard A. LaSalle, Brian Melancon, Kimberly McGhee, Leslie A. Lenert, Kathleen Brady

In the above article by He et al. [1], a funding source was missing. The following statement should have appeared at the end of the financial support section:

This publication was also supported in part by U54 GM104940 from the National Institute of General Medical Sciences of the National Institutes of Health, which funds the Louisiana Clinical and Translational Science Center.

The original article has been updated online to include this.
